# Comparison of the effect of Sunset yellow on the stomach and small intestine of developmental period of mice

**DOI:** 10.1016/j.heliyon.2024.e31998

**Published:** 2024-05-30

**Authors:** Erhan Şensoy

**Affiliations:** Karamanoglu Mehmetbey University Faculty of Health Science, Karaman, Turkey

**Keywords:** Food dyes, Mice, Gastrointestinal system, Growth

## Abstract

Sunset Yellow (SY), a synthetic food dye, is widely used in the food industry worldwide. The acceptable daily dosage for SY is 2.5 mg/kg/bw in humans. If SY is consumed in overdosage, it may cause histopathological effects in several organs. Studies in the literature about the effects of SY on growth and development in mammals are contradictory, and there are not enough of them. The investigation aims to determine SY's effects on the stomach and small intestine in different age groups of mice using histological methods. Control and treatment groups were created via mice aged 4, 8, and 10 weeks (n = 6). SY was administered by gavage at a level of 30 mg/kg/bw for 28 days to treatment groups. On the last day of the study, the mice were weighed and sacrificed by cervical dislocation. Stomach and small intestine tissues were removed from mice and transferred to 10 % formaldehyde. After passing through alcohol and xylene series and staining with Hematoxylin-Eosin, the tissues were evaluated under light and electron microscopy. The mean body weight (p = 0.01), mean stomach weight (p = 0.03), and mean small intestine weight were increased (p = 0.02) in treatment groups. In these groups, ruptures, fractures, and hemorrhage were detected in the small intestine tissue. In the stomach tissue, necrotic areas and hemorrhage were detected among the epithelial cells. The degenerations were more advanced in the weaning group. SY may be more harmful during weaning and puberty, but additional long-term studies are needed on the subject.

## 1 Introduction

Chemical compounds added to change the color of foods, prevent spoilage, and extend shelf life are known as food additives. Among these additives, food dyes are increasingly utilized. Sunset Yellow (SY) is an azo synthetic food dye employed to alter the color of foods [1]. SY is widely used in the food industry (soft drinks, desserts, confectionery, jams, chips, biscuits, ice creams), pharmaceuticals (children's medicines and syrups), and cosmetic products (soap, makeup, and skincare) worldwide [2]. The chemical formula of SY, which exhibits an orange-yellow hue, is C_16_H_10_N_2_Na_2_O_7_S_2_, with a molecular weight of 452.37 g/mol [3]. While the Acceptable Daily İntake (ADI) is reported as 4 mg/kg/bw [4], its use is restricted some countries such as Finland and Norway [5].

SY may induce hyperactivity, abdominal pain, nausea, vomiting, indigestion, and loss of appetite [6]. Long-term intake of SY in children has been associated with increased asthma symptoms due to histamine release [7]. Moreover, it was stressed that SY may cause retardation in growth and development parameters during childhood [8]. It undergoes metabolism in the intestinal wall similar to other azo dyes, leading to the production of free oxygen radicals and free aromatic amines in the liver. This process increases oxidative stress [9], potentially resulting in mutagenic effects [10,11]. Free radicals are known to play a crucial role in various pathological conditions such as cancer, cardiovascular disorders, arthritis, and liver diseases [6].

Numerous studies have investigated the effects of SY on different organs. It has been shown to induce histopathological changes in various parts of the brain [12], kidney tumors, and chromosome damage [13,14], as well as pathology in the liver and kidney in rats [15]. Additionally, SY has been reported to increase oxidative stress [16,17] and affect the homeostasis of the intestinal epithelium [18,19], potentially leading to pathologies in the intestinal epithelium with decreased defense power [20].

Research on the effects of SY in the gastrointestinal system primarily focuses on its metabolites in the colon, with insufficient studies investigating its effect on small intestinal epithelial cells. Therefore, determining the effect of SY on the stomach and small intestine epithelium and evaluating its reliability is crucial. This study aims to investigate the effect of SY on the stomach and small intestine epithelium and assess its reliability.

## 2 Material and method

### 2.1 Ethical permissions

Ethics committee permission was obtained from Selcuk University Experimental Medicine Research and Application Center (SUDAM) (July 24, 2020/2020-30).

### 2.2 Animals

Swiss Albino male mice (n:36) were obtained from Selcuk University Experimental Medicine Research and Application Center (SUDAM) and daily care of them was carried out in SUDAM throughout the study (mice stayed in standard housing condition, six mice were in per cage). Mice were kept at room temperature, subjected to a 12-h light-dark cycle, and were not subjected to food or water restriction. It is known that changing hormone levels in females depending on the menstrual cycle affects basic parameters such as growth and development. Male mice were preferred in the study to avoid potential alterations in the histological structure of the organs due to fluctuating hormone levels.

### 2.3 Groups

While creating the groups, it was aimed to adapt the developmental periods in human life to mice. For this purpose, three basic periods of human life (weaning, puberty, and adulthood) were taken as the basis. Groups were formed by proportioning these periods, taking into account the human lifespan and the mouse lifespan. The weaning period in humans begins at the 6th month, and in mice, it begins at the 28th day (4th week). Puberty begins at age 11.5 in humans and at the 8th week in mice. Adulthood begins at the age of 20 in humans and in the 10th week in mice [21,22]. Weaning (four-week-old), puberty (eight-week-old), and adulthood (ten-week-old) groups, as well as control groups, were created in mice (n = 6). SY (FD&C Yellow No. 6; purity 92 %) was given to the treatment groups by gavage for 28 days, but not to the control groups (Table 1).

**Table 1:** Groups and application.

Groups (n:6)	Age of Mice (weeks)	Chemical Substance	Chemical Substance Dosage	Applied Duration (Days)
**Group I**	4	SY	30 mg/kg	28
**Group II**	4	–	–	28
**Group III**	8	SY	30 mg/kg	28
**Group IV**	8	–	–	28
**Group V**	10	SY	30 mg/kg	28
**Group VI**	10	–	–	28

### 2.4 Histological evaluation

Tissues were fixed in 10 % formaldehyde, passed through alcohol and xylene series, and then transferred to paraffin. 6 μm thick sections taken from paraffin blocks were stained with Hematoxylin-Eosin [23]. For electron mixoscopy, tissues were coated with 4.39 nm iridium. The preparations were examined under a light microscope (Nikon DS Camera Control Unit DS-L1 and DS-5M digital camera at ×40 magnification) and an electron microscope (ZEISS Gemini SEM 500 Field Emission Scanning Electron Microscope; FE-SEM, 5K magnification).

Although the examination varies depending on the organ in histological evaluations, epithelial cell gaps and bleeding formation are known as the basic criteria [24,25]. For this reason, although organ-specific evaluations are made, the focus is on epithelial cell gaps and bleeding formation. Histomorphological differences between groups were taken into account in the examinations. In the stomach, as a histomorphological criterion, the general condition of the epithelium, spaces between epithelial cells, and hemorrhage formation were examined. In the small intestine, the general condition of the Lamina Muscularis (LM) layer and crypts, villus ruptures and fractures, and hemorrhage formation were examined [[26], [27], [28]].

### 2.5 Statistical analysis

Analysis of variance was applied to Mean body weight (first day)-Mean body weight (last day), Mean stomach weight, Mean small intestine weight, Relative stomach weight, and Relative intestine weight followed by an appropriate posthoc test (Duncan test) (p < 0.05) Statistical Package for Social Sciences (SPSS, version 21.0, IBM Corporation, Armonk, NY) software was used for all statistical analyses.

## 3 Results

### 3.1 Organ weights

Mice body weights were measured on both the first and last days of the study. On the 28th day, when the study was terminated, the animals were sacrificed by cervical dislocation. The stomach and small intestine weights were measured on the last day. Relative weights were obtained by dividing the average organ weight by the average body weight. An increase was determined in the average body (p: 0.02; p: 0.01), stomach (p: 0.03), and small intestine (p: 0.02) weights of the groups given SY. Similarly, an increase was detected in the relative stomach and relative small intestine values (Table 2).

**Table 2:** Average body, organ and relative organ values of mice (Mean ± S.E.).

	Mean body weight (first day)	Mean body weight (last day)	Mean stomach weight	Mean small intestine weight	Relative stomach weight	Relative intestine weight
**Group I**	11.15 **±** 0.10^a^	29.10 **±** 0.60^a^	0.210 **±** 0.18^a^	0655 ± 0.14^a^	0.0075^a^	0.0226^a^
**Group II**	11.23 **±** 0.21^a^	27.87 **±** 0.45^ab^	0.174 **±** 0.24^b^	0.627± 0.25^b^	0.0059^b^	0.0225^b^
**Group III**	28.04 **±** 0.80^b^	31.60 **±** 1.07^a^	0.296 **±** 0.49^ab^	0.567 ± 0.22^bc^	0.0094^ab^	0.0207^bc^
**Group IV**	28.55 **±** 0.76^b^	31.28 **±** 0.70^ab^	0.286 **±** 0.13^b^	0.610 ± 0.16^b^	0.0090^b^	0.0195^b^
**Group V**	31.10 **±** 1.40^c^	35.67 **± 1**.49^a^	0.303 **±** 0.12^ac^	0.703 ± 0.47^ac^	0.0085^ac^	0.0202^ac^
**Group VI**	31.36 **±** 1.37^c^	34.83 **±** 1.60^ab^	0.252 **±** 0.17^c^	0.520 ± 0.25^b^	0.0072^c^	0.0146^b^
**Sig.**	0.02	0.01	0.03	0.02		

S.E: Represents standard error. Values shown with different letters differ from each other at the *p* < 0.05 level.

### 3.2 Histological evaluation

#### 3.2.1 Stomach tissue

**Fig. 1:**
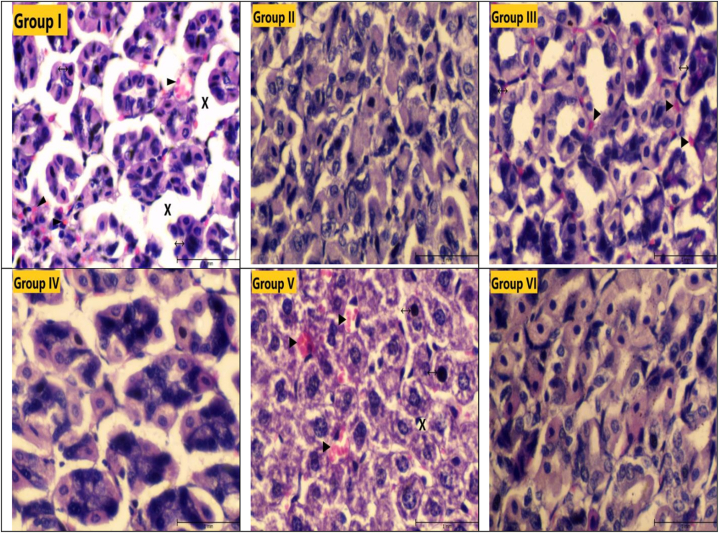
Light microscope images of stomach tissue (HE staining, Magnification line: 100 mm X 40). ►: Hemorrhage**, ↔:** Darkly stained nucleus, **X:** Spaces between epithelial cells.

**Fig. 2:**
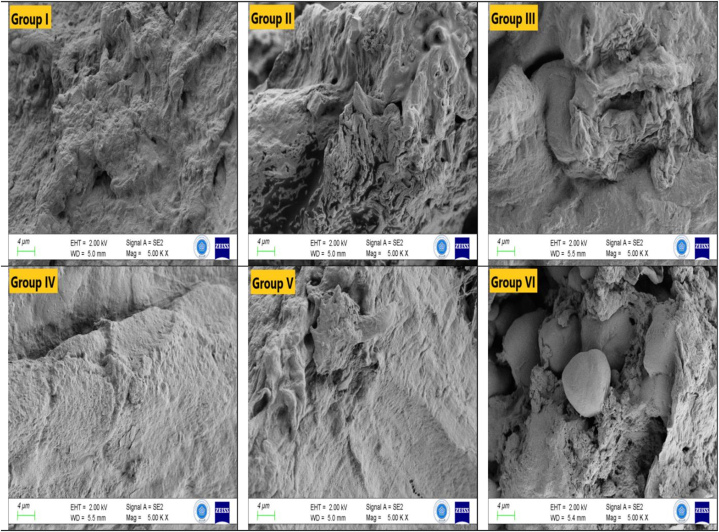
Electron microscope images of stomach tissue (EHT: 2.00 kV; Magnification: 5.00 KX).

In the control groups (second, fourth, and sixth groups), the stomach tissue had a normal histological appearance, and its contours were regular. While the cytoplasm and nucleus were stained in normal colors, no gaps or hemorrhages were found between the epithelial cells. In the first, third, and fifth groups, dark staining of the nucleus and cytoplasm, gaps between epithelial cells, and hemorrhage were detected. The gaps between epithelial cells, interpreted as mucosal degeneration, were mostly in group one. The nucleus and cytoplasm were stained the darkest in group third. The presence of intense hemorrhage in group first and group fifth was considered important (Fig. 1, Fig. 2).

#### 3.2.2 Small intestine tissue

**Fig. 3:**
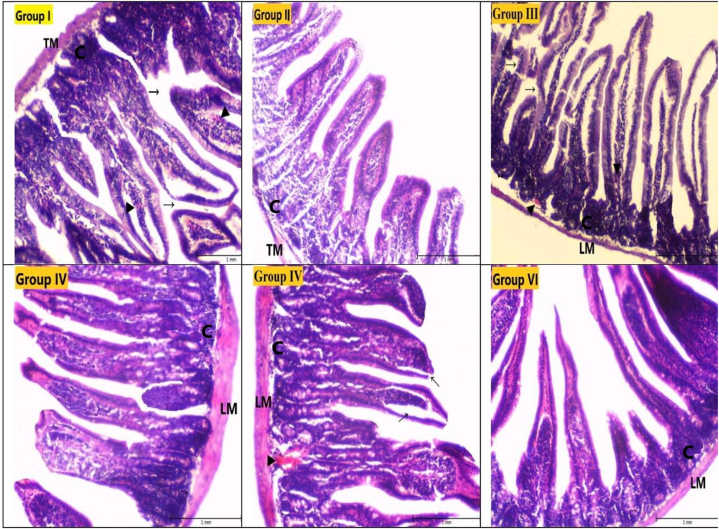
Light microscope images of the small intestine tissue (HE staining, Magnification line: 100 mm X 40), TM: Tunica Muscularis, **LM:** Lamina Muscularis, **C:** Cript cell, **→:** Rupture in villi, **►:** Hemorrhage.

**Fig. 4:**
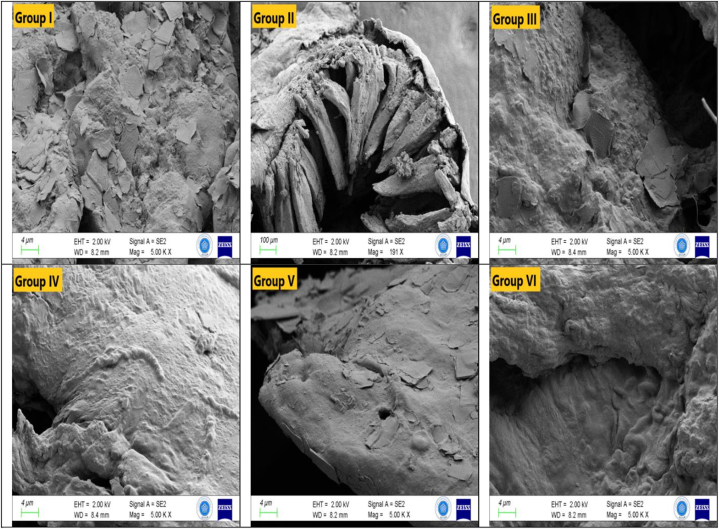
Electron microscope images of small intestine (EHT: 2.00 kV; Magnification: 5.00 KX).

In the control groups (second, fourth, and sixth groups), the small intestine tissue had a normal histological appearance. The contours of the Tunica Muscularis (TM) layer were smooth, and the general histological structure was normal. The crypts and villi maintained their structural integrity, and no degenerations such as tears or fractures, necrotic areas, or bleeding were detected in the villi in the control groups. In the first group, although the contours of the TM layer were smooth, the crypts could not maintain their structural integrity. Ruptures and fractures in the villi and hemorrhage were detected. In the third group, the TM layer became very thin, and the structural integrity of the TM and crypts was impaired. Gaps were seen between the crypts, indicating necrosis. Additionally, ruptures, fractures, and hemorrhage in the villi were detected. In the fifth group, although the contours of the TM layer were determined to be smooth and the structural integrity of the crypts was determined, extensive hemorrhage was observed. Additionally, severe ruptures and fractures in the villi were considered important (Fig. 3, Fig. 4).

## 4 Discussion

Sunset Yellow, an azo synthetic dye, may affect the intestinal epithelium and induce changes in intestinal secretion. Moreover, it can disrupt intestinal signaling interactions by exerting an antagonist effect on the Glucagon Like Peptide-1 (GLP-1) receptor, a peptide hormone [29]. Investigations regarding the relationship between food additives and obesity have indicated that food additives increase the risk of obesity [5,30,31]. According to the results of our study, the average body weight of mice with SY added to their diets increased statistically significantly compared to the controls. Our results are consistent with the study in which a significant increase in body weight of 42-day-old male Wistar rats fed SY (10 mg/kg/body weight) for two weeks [32] and accordance with other studies [[33], [34], [35]]. The increase may indicate an obesity risk, aligning with findings from similar studies. On the other hand, it has been reported that a decrease in body weight gain was observed in mice fed SY (4 mg/kg/bw) orally for 40 days [36]. According a smilar investigation, the average weight gain of male mice fed orally with SY (6.17 mg/kg/bw) for 12 weeks was similar to the control group [37]. The fact that our results are not similar to the findings of these studies may be due to the different age, gender, SY dose and application time of the mice used in the studies.

The effects of food dyes on the physiology of the gastrointestinal system and whether they cause pathology in organs such as the stomach and small intestine are issues that have recently attracted the attention of researchers. However, the current literature on the pathologies caused by food dyes in these organs is quite limited and contradictory. It has been stressed that food dyes containing sulfonate groups may irritate the gastrointestinal system [38]. It has been emphasized that SY may negatively affect digestive physiology by inhibiting pepsin activity [39]. Ulcerative lesions and bleeding were observed in the stomach of rats given orally at different doses of SY [40]. According a smilar investigation, hyperplasia was detected in the stomach of mice fed with SY (30 mg/kg/bw) [41]. It has been reported that in mice fed SY (30mg/kw/bw) the number of degenerative cells increased in the liver tissue, and lymphocytic infiltration increased in the pancreatic tissue [42]. Histopathologies such as lesions, vacuoles, and the presence of parietal cells with irregular nuclei have been reported in the gastric mucosa of male albino rats fed with Tartrazine (200 mg/kg/bw), which is from the azo dye group like SY [43]. Additionally, it has been reported that chocolate brown dye (200 mg/kg/bw), another azo dye, causes histopathology in the stomach and intestines of rats [44]. Furthermore, malachite green dye has been reported to cause necrosis and induce apoptosis in the stomach cell line [45]. Our results, which can be summarized as lesions and bleeding in the gastric mucosa and indicating mucosal degeneration, are compatible with similar studies.

In contrast, it has been emphasized that SY caused limited pathological effects in the stomach tissue of rats fed SY for 30 and 60 days [46]. According a similar study, SY did not cause pathology in the stomach tissue of mice but caused a partially toxic effect [47]. It has been reported that SY did not cause pathology in the stomach cells of mice to which SY (2000 mg/kg/bw) and some other food coloring mixtures were added to their diets [48]. In a similar study, it has been stated that no histopathological change was observed in the stomach tissue of 42-day-old male rats fed with SY (10 mg/bw) for two weeks [32]. The authors reported that this may be due to the azo bonds of food dye being stable under the acidic conditions of the stomach. It has been stated that no histopathological effects were observed in the stomach of male or female rats fed a mix of SY, tartrazine, and amaranth [49]. According a smilar investigation no histopathological findings were found in the internal organs of rats fed SY (7 mg/kg/bw) for 30 days [50]. It is thought that the reason why our results are not compatible with the studies that concluded that SY does not cause histopathological changes in the stomach may be due to the difference in the SY dose and application period used in other studies.

It has been reported that SY, which acts through dysbiosis of the gastrointestinal microbiota, may pose a risk in triggering inflammatory bowel diseases such as colitis [51]. The authors reported that a food coloring mixture including SY may be a risk factor for the development of colitis in mice. In an observational study conducted in pediatric individuals with inflammatory bowel disease, dietary elimination of certain triggers, such as SY, was shown to be effective in the treatment of lesions [52]. A recent study reported that consumption of artificial food additives such as SY may affect the intestinal microbiota and disrupt the intestinal barrier [53]. The authors pointed out that this condition can lead to chronic inflammation and has a high risk of potentially accelerating the development of ulcerative colitis. Current studies indicate that SY-induced oxidative stress causes inflammation [54] and plays an important role in pathogenesis [[55], [56], [57]]. Oxidative stress can disrupt intestinal epithelial homeostasis, causing inflammation and inhibiting proliferation [5]. According a investigation, a significant increase in the number of white blood cells due to acute inflammation was observed in rats fed SY [58]. In a similar study, intestinal inflammation and necrosis were noted in male mice orally fed SY (6.17 mg/kg/bw) for 12 weeks [37]. It has been reported that Green Leaf Color, a type of food dye, causes necrosis, vacuole formation, and mucosal disruption in mouse villi in rat [59]. It has been reported that SY (2.5 mg/kg) added to fertile chicken embryos causes advanced degranulation of mucosal mast cells [13]. This situation, which is compatible with the inflammation we detected in the small intestinal mucosa, is thought to be a result of the change caused by SY in the intestinal microbiota.

Regeneration of intestinal epithelial cells relies on proliferative progenitors located at the crypt base, where cells divide intensively [60]. Crypts, which are critical in intestinal epithelial development [61], have a synergistic effect on epithelial proliferation and differentiation [62,63]. Advanced intestinal damage and crypt degeneration have been reported in mice fed SY (40 mg/kg/bw) [5]. It has been reported that hemorrhage and lesions were detected in the crypts of mice fed SY (30 mg/kg/bw) [41]. In a similar study, it was reported that significant pathologies such as desquamation, degeneration in the crypts, cell infiltration, and lymphoid hyperplasia in the lamina propria were observed in the small intestine of 42-day-old male Wistar rats fed SY (10 mg/kg/bw) for two weeks [32]. On the other hand, it has been reported that SY does not cause pathology in the mouse small intestine but has a partial toxic effect [47]. According a similar study, no histopathological effects were observed in the small intestine of male or female rats fed mix of SY, tartrazine, and amaranth for 64 weeks [49]. It was reported that no genotoxic effect was observed on the intestinal cells of mice fed SY (2000 mg/kg/bw) two times [38]. Our findings indicating hemorrhage in the crypts, disruption of crypt integrity, and intercrypt necrosis are compatible with the results of with many part of the previous studies. According to the general histological evaluation of the stomach and small intestine, it is thought that the histopathologies determined are caused by the degradation products of SY.

The investigation has limitations such as the sample consisting of 36 animals (n = 6), the animal model comprising male mice, and the administration of the only the same food dye (SY) to animals in all age groups. As avenues for future research to overcome these limitations, it may be beneficial to increase the number of animals in each group. It may also be recommended that groups be composed of equal numbers of male and female individuals. Additionally, giving combinations of SY and other azo dyes to experimental groups and determining which dye/dye combination is more harmful stand out as potential issue that need to be investigated in future studies on the subject.

## 5 Conclusion

Since organ development continues during childhood in addition to intrauterine life, an interruption in the development process of an organ is likely to cause the individual to face different health problems in the future. Based on the findings of the study, it is recommended that policymakers enact legal measures for the production and sale of foods containing SY. It may be effective if food producers comply with these laws meticulously and are inspected by authorized institutions. Parents are advised to limit their children's consumption of foods containing food dyes.

## Ethics permission

Ethics committee permission was obtained from Selcuk University Experimental Medicine Research and Application Center (SUDAM) (July 24, 2020/2020-30). We declerated that the study was carried out in accordance with either the U.K. Animals (Scientific Procedures) Act, 1986 and associated guidelines, the European Communities Council Directive 2010/63/EU, or the National Institutes of Health – Office of Laboratory Animal Welfare policies and laws and the study comply with the ARRIVE guidelines.

## Financial Support

N/A.

## Data availability statement

Supplementary material related to this article can be found online at https://docs.google.com/document/d/1y9p_ws780QIzD3SRSgouuD9lEhyY0pzE/edit.

## CRediT authorship contribution statement

**Erhan Şensoy:** Writing – review & editing, Writing – original draft, Visualization, Validation, Supervision, Software, Resources, Project administration, Methodology, Investigation, Funding acquisition, Formal analysis, Data curation, Conceptualization.

## Declaration of competing interest

The authors declare that they have no known competing financial interestsor personal relationships that could have appeared to influence the work reported in this paper.
